# A high functional cure rate was induced by pegylated interferon alpha-2b treatment in postpartum hepatitis B e antigen-negative women with chronic hepatitis B virus infection: an exploratory study

**DOI:** 10.3389/fcimb.2024.1426960

**Published:** 2024-08-08

**Authors:** Wenting Zhong, Lanzhi Yan, Yage Zhu, Lei Shi, Yingli He, Tianyan Chen, Jie Zheng

**Affiliations:** ^1^ Department of Infectious Disease, the First Affiliated Hospital of Xi’an Jiaotong University, Xi’an, Shaanxi, China; ^2^ Department of Radiology, the First Affiliated Hospital of Xi’an Jiaotong University, Xi’an, Shaanxi, China

**Keywords:** postpartum therapy, functional cure, advantageous population, HBsAg loss, Peg-IFN alpha, postpartum women

## Abstract

**Background and aims:**

Limited data have been reported on achieving functional cure using pegylated interferon (Peg-IFN) alpha-2b treatment for postpartum hepatitis B e antigen (HBeAg)-negative women with chronic hepatitis B virus (HBV) infection. This study was to assess the effectiveness and safety of Peg-IFN alpha-2b in HBV postpartum women without HBeAg and identify factors linked to the functional cure.

**Methods:**

A total of 150 HBeAg-negative postpartum women were retrospectively recruited.47 patients received Peg-IFN alpha-2b [Peg-IFN(+) group] and 103 patients did not [Peg-IFN(-) group]. Propensity score matching (PSM) was used to adjust the baseline imbalance between the two groups. The patients were followed for at least 48 weeks. The primary endpoints were hepatitis B surface antigen(HBsAg) loss and HBsAg seroconversion at 48 weeks. Logistic regression analysis was used to assess factors associated with HBsAg loss at 48 weeks.

**Results:**

At week 48,the HBsAg loss and seroconversion rate in Peg-IFN(+) group were 51.06%(24/47) and 40.43%(19/47), respectively. Even after PSM, Peg-IFN(+) group still showed higher HBsAg loss rate (50.00% vs 7.14%,p<0.001) and higher HBsAg seroconversion rate (38.10% vs 2.38%,p<0.001). Baseline HBsAg levels (Odds Ratio [OR]: 0.051, 95% Confidence Interval [CI]: 0.003-0.273, P=0.010), HBsAg at week 24 (OR:0.214, 95%CI:0.033-0.616, P=0.022), HBsAg decline at week 24 (OR:4.682, 95%CI: 1.624-30.198, P=0.022) and postpartum flare (OR:21.181, 95%CI:1.872-633.801, P=0.030) were significantly associated with HBsAg loss at week 48 after Peg-IFN alpha-2b therapy. Furthermore, the receiver operating characteristic curve (ROC) showed that the use of baseline HBsAg<182 IU/mL, HBsAg at week24 < 4 IU/mL and HBsAg decline at week24>12IU/mL were good predictors of HBsAg loss. No serious adverse events were reported.

**Conclusion:**

Peg-IFN alpha-2b treatment could achieve a high rate of HBsAg loss and seroconversion in HBeAg-negative postpartum women with reliable safety, particularly for patients experience postpartum flare and have low baseline HBsAg levels.

## 1 Introduction

In 2022, about 3.2% of the global population had chronic hepatitis B virus (HBV) infection (Polaris Observatory Collaborators, 2023). HBV is the primary cause of cirrhosis and hepatocellular carcinoma (HCC) worldwide (Hsu et al., 2023).

“Functional cure” has become the paramount therapeutic objective for chronic HBV infection, aided by medical advancements (Hou et al., 2017),which denotes hepatitis B (HBsAg) loss in chronic hepatitis B (CHB) patients, with or without hepatitis B surface antibodies (HBsAb).While covalently closed circular DNA(cccDNA) may persist in the liver, there is an amelioration of liver inflammation and histopathology, markedly reducing end-stage liver disease risk. However, spontaneous HBsAg reversal is exceedingly rare, occurring at a rate of roughly 1% annually (Zhou et al., 2019). Even with long term use of nucleos(t)ide analogues (NAs), annual HBsAg clearance rate is still below 2% (Levrero et al., 2018; Tang et al., 2018; Terrault et al., 2018; Wu et al., 2019).

Peg-IFN alpha possesses immunomodulatory and antiviral properties, making a functional cure more achievable. However, responses differ among individuals, and its effectiveness is notable in only a specific group of patients, often referred to as the advantaged population (Wieland et al., 2005; Sadler and Williams, 2008; Belloni et al., 2012). Several studies (Chinese Society of Infectious Disease Chinese Society of Hepatology, and Chinese Medical Association, 2019; De Ridder et al., 2021; Ren et al., 2021; Huang et al., 2022; Wang et al., 2022a) have suggested that individuals who are HBeAg-negative with HBsAg levels below 3000 IU/ml, and particularly below 1500 IU/ml, are more likely to attain a functional cure with Peg-IFN alpha.

Concerning HBeAg-negative mothers with a low HBsAg level and low HBV DNA level, the postpartum period’s significant hormonal changes and immune system restructuring influence HBV infection’s natural course (Carr et al., 1981; Lin et al., 1993; Wegmann et al., 1993; Marzi et al., 1996; Elenkov, 2004; Groer et al., 2015). However, current guidelines for pregnant women with HBV concentrate on mother-to-child transmission (MTCT), and there is debate about postpartum treatment approaches.

Prior studies have observed that replacing telbivudine with Peg-IFN alpha-2a alongside adefovir treatment postnatally, when postnatal alanine aminotransferase (ALT) levels exceeded twice the upper limit of normal(ULN), leads to HBeAg titer decreasing by over 20% from baseline, resulting in a 56.7% HBeAg seroconversion rate and a 26.7% HBsAg loss rate (Lu et al., 2015).This rate surpasses or matches that of the general chronic HBV population during immune activation (Lau et al., 2005; Buster et al., 2008; Marcellin et al., 2016). Two small studies (Guo et al., 2023; Han et al., 2021) from the American Association for the Study of Liver Diseases (ASALD), which are still ongoing, have found that Peg-IFN can achieve HBsAg loss rates of over 40% in postpartum women with low HBsAg levels. Additionally, other studies (Liu et al., 2017; Feng et al., 2023) have demonstrated that regardless of pregnancy ALT levels, continuing oral NAs monotherapy for antiviral therapy in the postpartum period aids in reducing HBsAg and HBeAg levels. This treatment yields an HBeAg seroconversion rate of approximately 10-15%, making it markedly more effective than it for the general chronic HBV population during the immune-tolerant phase.

Thus, the postpartum phase presents an opportune time for chronic HBV treatment and disease regression. HBeAg-negative postpartum mothers may have a heightened likelihood of achieving a functional cure with Peg-IFN alpha compared to other HBeAg-negative patients. However, most prior studies on the functional cure with Peg-IFN have excluded postpartum women due to the special physiological state of the postpartum period and the difficulty of postpartum follow-up and management.

This study aimed to assess the therapeutic efficacy and safety of Peg-IFN alpha-2b in achieving a functional cure rate for chronic HBV infection in postpartum HBeAg-negative women.

## 2 Method

### 2.1 Study design

This retrospective cohort study enrolled 150 HBV postpartum women between January 2018 and December 2022 at the First Affiliated Hospital of Xi’an Jiaotong University, China. The patients were categorized into two groups based on Peg-IFN alpha-2b treatment: 47 received Peg-IFN alpha-2b (the Peg-IFN (+) group) and 103 did not (the Peg-IFN (-) group). Postpartum women who did not receive Peg-IFN alpha-2b were monitored at least every 24 weeks like other HBV-infected patients, while those who received Peg-IFN alpha-2b were monitored at least every 12 weeks. The inclusion criteria were: 1) postpartum women (12-72 weeks post-delivery) with chronic HBV infection, HBsAg positive for ≥6 months, and HBsAg ≤ 3000IU/mL at baseline; 2) HBeAg negative at baseline; 3) HBV DNA ≤ 2000IU/ml at baseline; 4) followed up for ≥48 weeks. The exclusion criteria were: 1) co-infection with hepatitis C, hepatitis D, or HIV; 2) alcoholic liver disease or autoimmune liver disease; 3) decompensated cirrhosis or HCC; 4) undergoing radiotherapy, chemotherapy, or immunosuppressive therapy; 5) serum creatinine levels <50 ml/min/1.73 m^2^; 6) mental disorders; 7) other contraindications for Peg-IFN alpha-2b use.

This study received approval from the Ethics Committee of the First Affiliated Hospital of Xi’an Jiaotong University (XJTU1AF2024LSK-2021-464) and adhered to the 1975 Declaration of Helsinki, clinical practice guidelines (Chinese Society of Infectious Diseases, Chinese Medical Association; Chinese GRADE Center, 2019; Kumar et al., 2022), and local regulatory requirements. Written informed consent was obtained from all patients regarding their participation in the study.

### 2.2 Primary outcome and secondary outcomes

The primary outcome was the rate of HBsAg loss and seroconversion at 48 weeks. HBsAg loss was defined as HBsAg levels dropping below 0.05 IU/mL, with or without the emergence of HBsAb. HBsAg seroconversion was defined as HBsAg levels dropping below 0.05 IU/mL, accompanied by HBsAb levels exceeding 10 mIU/mL.

Secondary outcomes included: (1) the rate of HBsAg loss and seroconversion at 24 weeks; (2) reduction in HBsAg levels from baseline to 24 weeks and 48 weeks; (3) rate of HBV DNA undetectability at 24 weeks and 48 weeks. HBV DNA undetectability was defined as HBV DNA levels below 20 IU/mL.

### 2.3 Other measurements

Clinical data and laboratory test results were retrieved from the Electronic Medical Records (EMR) system. The clinical data included age, NAs usage, occurrence of postpartum flare (ALT>2 ULN within 12 weeks post-delivery, ULN=40 U/L), time from delivery to study enrollment, and HBsAg reduction from mid-pregnancy to delivery. The laboratory test results comprised a routine blood test, liver function and hepatitis B-related markers. The safety assessment included systemic symptoms, skin symptoms, gastrointestinal symptoms, blood tests and endocrine tests.

### 2.4 Antiviral regimens

Patients previously treated with NAs during pregnancy continued NAs post-delivery, with the option to add Peg-IFN alpha-2b therapy (Xiamen Amoytop Biotech Co, LTD, Xiamen, China, 135μg, subcutaneous injection, once weekly) for a minimum of 48 weeks based on patient preference. The NAs treatment used was tenofovir disoproxil fumarate. Those not treated with NAs during pregnancy either underwent postpartum monitoring alone or chose to add Peg-IFN alpha-2b therapy for a minimum of 48 weeks at their discretion. Patients could stop the treatment before 48 weeks after they achieved HBsAg seroconversion. The median course of the Peg-IFN alpha-2b therapy was 48 (range: 36 – 80) weeks.

### 2.5 Propensity score matching method

**Figure 1:**
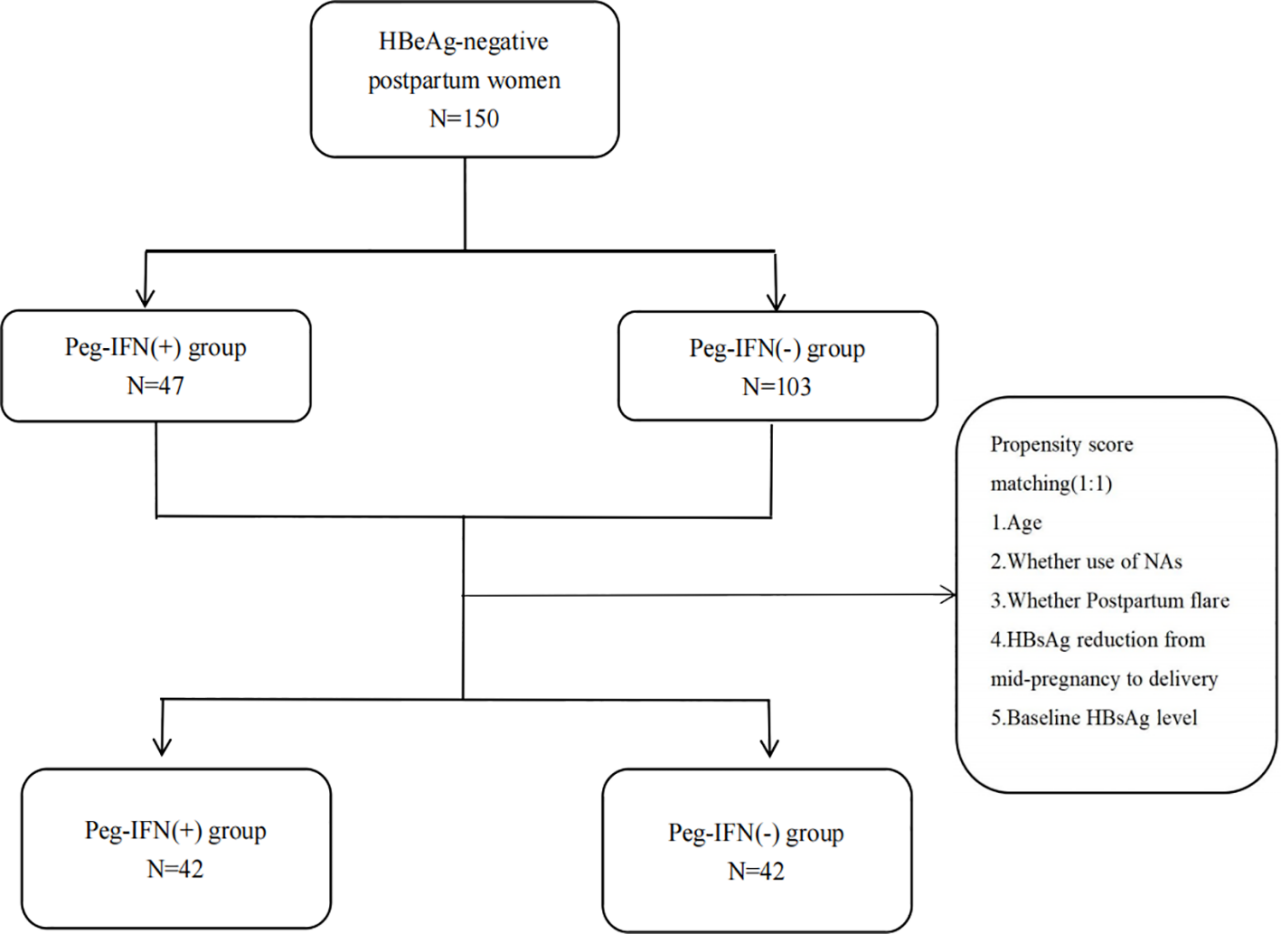
Flow diagram illustrating the selection of the study population.

The PSM method was employed to match the Peg-IFN (-) group to the Peg-IFN (+) group at a 1:1 ratio based on the following covariates: age, occurrence of postpartum flare, NAs usage, HBsAg reduction from mid-pregnancy to delivery, and baseline HBsAg level, aiming to address imbalances in confounding factors in the retrospective cohort study (**Figure 1**). The R package “matchit” facilitated the one-to-one matching process.

### 2.6 Statistical analysis

Before PSM, continuous variables with a normal distribution were expressed as means ± standard deviations (SDs) and compared between groups using t-tests. For continuous variables not following a normal distribution, median (interquartile range [IQR]) values were presented., and group differences were assessed using the Mann-Whitney U-test. Categorical variables were presented as the number of patients (with corresponding proportions) and differences between groups were evaluated using the Chi-squared test or Fisher’s exact test.

After PSM, paired t-tests or Signed Wilcoxon tests were utilized to compare continuous variables between the pair-matched groups. The McNemar test was employed to compare categorical variables between the matched groups. Univariable and multivariable logistic regressions were used to identify the factors independently associated with HBsAg loss within the Peg-IFN (+) group. Odds ratios (OR) and the 95% confidence interval (95% CI) were reported for the factors. For continuous predictors, the receiver operating characteristic (ROC) curve analyses were used to determine the optimal cut-off values by utilizing the Youden index. A p-value < 0.05 was considered statistically significant. All statistical analyses were performed using SPSS version 29.0 software (IBM, Armonk, NY, USA) and R software (version 3.6.3).

## 3 Results

### 3.1 Baseline characteristics of patients enrolled in the study

The baseline characteristics of patients before and after PSM are presented by group in **Table 1**. Prior to PSM, age and baseline HBsAg (log10 IU/mL) showed significant differences between the two groups (P<0.05). The median onset time of Peg-IFN alpha-2b therapy for the Peg-IFN (+) group was 11.93 (8.27 to 14.37) months. Following PSM, 42 patients in the Peg-IFN(-) group were matched one-to-one with patients in the Peg-IFN(+) group based on age, postpartum flare, NAs usage, HBsAg reduction from mid-pregnancy to delivery, and the baseline HBsAg level. Subsequently, no statistical differences were observed in baseline characteristics between the two groups after PSM.

**Table 1:** Clinical characteristics of patients before and after PSM.

Variables	Before PSM			After PSM		
	Peg-IFN (-) N=103	Peg-IFN (+) N=47	P	Peg-IFN (-) N=42	Peg-IFN (+) N=42	P
**Use of NAs (%)**	64 (62.14)	28 (59.57)	0.765	28 (66.67)	27 (64.29)	1.000
**Age (years)**	29.80 ± 3.56	31.49 ± 3.38	0.007	31.43 ± 3.74	31.52 ± 3.49	0.891
**Postpartum flare, N (%)**	13 (12.62)	11 (24.44)	0.073	16 (38.10)	15 (35.71)	1.000
**Time from delivery to study enrollment (months)**	11.30 (7.80,14.43)	11.93 (8.27,14.37)	0.741	11.97 (8.10,14.70)	12.17 (8.10,14.37)	0.960
**ALT (U/L)**	18.96 ± 8.08	17.87 ± 8.90	0.465	19.18 ± 7.55	17.62 ± 9.06	0.400
**AST (U/L)**	21.36 ± 6.17	20.94 ± 8.30	0.732	21.65 ± 5.37	20.31 ± 6.35	0.302
**HBsAg (log10 IU/mL)**	2.98 (2.38,3.22)	2.55 (1.95,2.99)	0.006	2.69 (2.09,3.02)	2.55 (1.45,3.01)	0.288
**HBV DNA (log10 IU/mL)**	1.30 (1.30,2.00)	1.30 (1.30,1.30)	0.107	1.30 (1.30,2.00)	1.30 (1.30,1.30)	0.527
**HBsAg reduction from mid-pregnancy to delivery (log10 IU/mL)**	0.05 (-0.07,0.21)	0.09 (-0.04,0.52)	0.087	0.07 (-0.04,0.25)	0.09 (-0.04,0.52)	0.339

PSM, propensity score matching; Peg-IFN alpha-2b, pegylated interferon alpha-2b; NAs, nucleos (t)ide analogs; AST, aspartate aminotransferase; ALT, alanine aminotransferase; HBV, hepatitis B virus; HBsAg, hepatitis B surface antigen.

P<0.05 was considered statistically significant.

### 3.2 Outcomes before and after PSM

Before PSM, the Peg-IFN (+) group achieved both higher HBsAg loss rate (week 24: 29.79% vs. 0.97%, P<0.001; week 48: 51.06% vs. 2.91%, P<0.001) and HBsAg seroconversion rate (week 24: 21.28% vs. 0.97%, P<0.001, week 48: 40.43% vs. 0.97%, P<0.001). Please refer to **Table 2**.

**Table 2:** Outcomes for the Peg-IFN (-) group and Peg-IFN (+) group before and after PSM.

Variables	Before PSM			After PSM		
	Peg-IFN (-) N=103	Peg-IFN (+) N=47	P	Peg-IFN (-) N=42	Peg-IFN (+) N=42	P
Week24						
**HBsAg reduction from baseline, log10 IU/mL**	0.03 (-0.10,0.14)	1.08 (0.53,2.55)	<0.001	0.04 (-0.07,0.25)	1.03 (0.47,2.69)	<0.001
**HBsAg loss, n (%)**	1 (0.97)	14 (29.79)	<0.001	1 (2.38)	13 (30.95)	<0.001
**HBsAg seroconversion, n (%)**	1 (0.97)	10 (21.28)	<0.001	1 (2.38)	9 (21.43)	0.008
**HBV DNA undetectable, n (%)**	67 (65.05)	38 (80.85)	0.050	30 (71.43)	34 (80.95)	0.454
Week48						
**HBsAg reduction from baseline, log10 IU/mL**	0.07 (-0.09,0.21)	1.85 (1.13,3.25)	<0.001	0.07 (-0.09,0.24)	1.61 (1.07,3.21)	<0.001
**HBsAg loss, n (%)**	3 (2.91)	24 (51.06)	<0.001	3 (7.14)	21 (50.00)	<0.001
**HBsAg seroconversion, n (%)**	1 (0.97)	19 (40.43)	<0.001	1 (2.38)	16 (38.10)	<0.001
**HBV DNA undetectable, n (%)**	67 (65.05)	45 (95.75)	<0.001	30 (71.43)	40 (95.24)	0.013

PSM, propensity score matching; Peg-IFN alpha-2b, pegylated interferon alpha-2b; HBV, hepatitis B virus; HBsAg, hepatitis B surface antigen.

P<0.05 was considered statistically significant.

After PSM, compared to patients in the Peg-IFN (-) group, those in the Peg-IFN (+) group exhibited greater HBsAg reduction (1.03 [0.47, 2.69] vs. 0.04 [-0.07, 0.25] log10 IU/mL, p < 0.001), a higher rate of HBsAg loss (30.95% vs. 2.38%, p < 0.001), and a higher rate of HBsAg seroconversion (21.43% vs. 2.38%, p = 0.008) at week 24. No significant difference was observed in the rate of HBV DNA undetectability at week 24.

**Figure 2:**
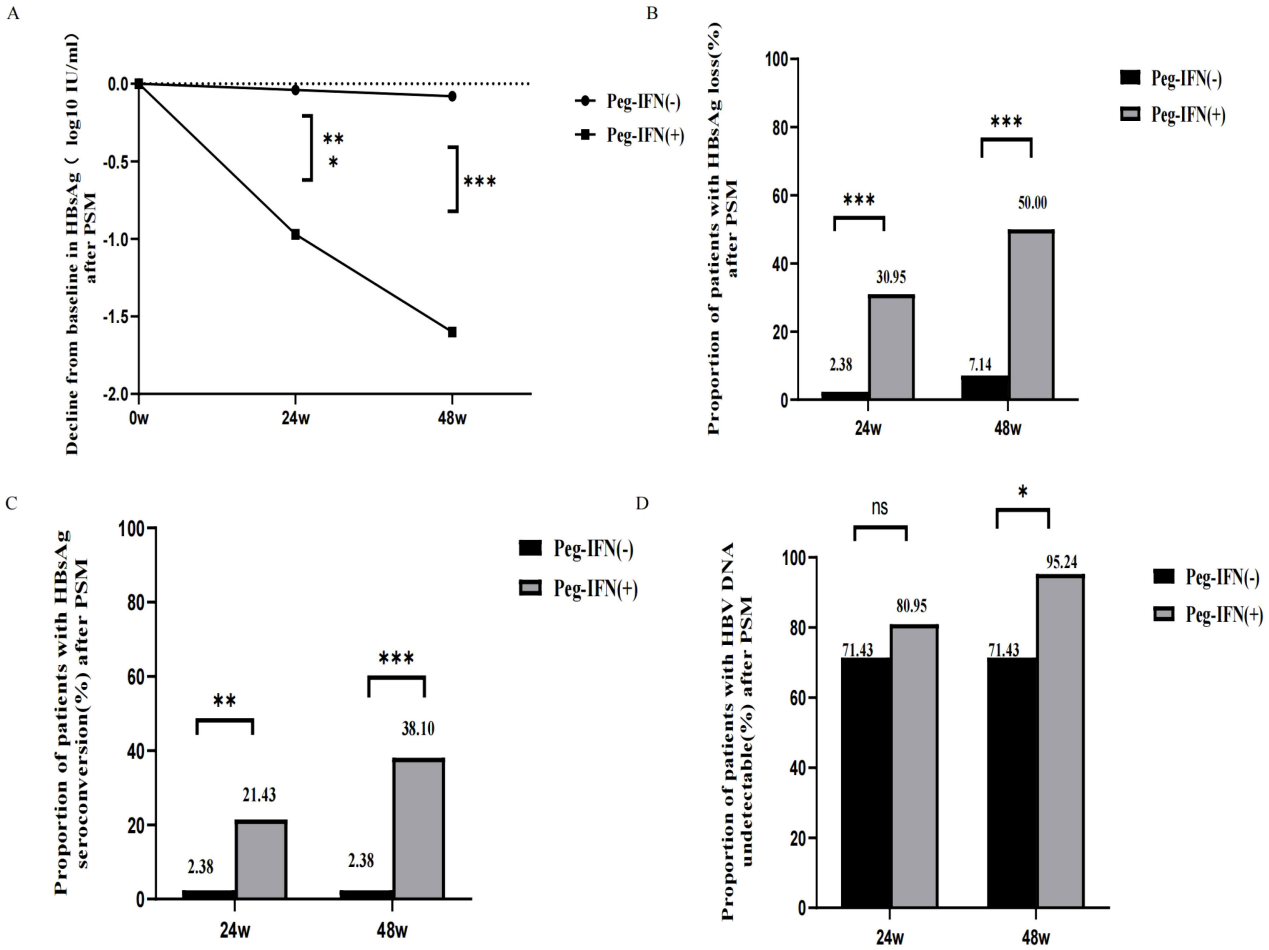
Outcome after PSM. **(A)** Decline from baseline in HBsAg after PSM. **(B)** HBsAg loss rates in week 24 and week 48 after PSM. **(C)** HBsAg seroconversion rates in week 24 and week 48 after PSM. **(D)** HBV DNA undetectable rates in week 24 and week 48 after PSM. NS, non-significant (p > 0.05), (*P < 0.05, **P <0.01, ***P < 0.001).

At week 48, the disparities in the HBsAg reduction (1.61 [1.07,3.21] vs. 0.07 [-0.09,0.24] log10 IU/mL, p < 0.001), rate of HBsAg loss (50.00% vs. 7.14%, p < 0.001), and seroconversion (38.10% vs. 2.38%, p < 0.001) were even more pronounced. Additionally, a higher proportion of patients in the Peg-IFN (+) group achieved HBV DNA undetectability at week 48 (95.24% vs. 71.43%, p = 0.013). Please refer to **Figure 2**.

### 3.3 Predictors of functional cure

The results of univariate and multivariate analyses for factors associated with HBsAg loss are presented in **Table 3A**. In the univariate analyses, a lower HBsAg level at baseline (OR:0.043, 95% CI: 0.007-0.276, P = 0.003), a lower HBsAg level at week 12 (OR:0.188, 95% CI:0.076-0.462, P <0.001), a greater HBsAg decline at week 12(OR:4.283, 95% CI:1.511-12.141, P = 0.006), a lower HBsAg level at week 24 (OR:0.161, 95% CI:0.063-0.413, P <0.001), a greater HBsAg decline at week 24 (OR:4.202, 95% CI:1.885-9.368, P <0.001),postpartum elevated ALT(>ULN)(OR:4.320, 95% CI: 1.179-15.827, P =0.027) and postpartum flare (OR:7.269, 95% CI:1.353-39.045, P =0.021) were associated with a higher likelihood of HBsAg loss after 48- weeks of Peg-IFN alpha-2b therapy. Multivariate logistic regression revealed that the HBsAg level at baseline (OR:0.051, 95% CI:0.003-0.273, P = 0.010), HBsAg level at week 24 (OR:0.214, 95% CI: 0.033-0.616, P = 0.022), HBsAg decline at week 24 (OR:4.682, 95% CI: 1.624-30.198, P = 0.022) and postpartum flare (OR:21.181, 95% CI: 1.872-633.801, P = 0.030) remained associated with a higher likelihood of HBsAg loss after 48 weeks of Peg-IFN alpha-2b therapy.

**Table 3:** (A) The predictive factors for HBsAg loss at week 48 in Peg-IFN-treated patients and (B) prediction of HBsAg loss at week 48 in patients who received Peg-IFN treatment.

Predictors (A)	Univariate analysis			Multivariate analysis		
	OR	95%CI	P	OR	95%CI	P
**use of NAs**	0.900	(0.280,2.888)	0.859			
**Age,years**	0.950	(0.800,1.129)	0.562			
**Weight**	1.057	(0.973,1.147)	0.189			
**BMI**	1.126	(0.892, 1.423)	0.318			
**Postpartum elevated ALT(>ULN)**	4.320	(1.179,15.827)	0.027	5.849	(0.770,64.302)	0.103
**Postpartum flare(ALT>2ULN)**	7.269	(1.353,39.045)	0.021	21.181	(1.872,633.801)	0.030
**Time from delivery to study enrollment (months)**	0.936	(0.802,1.092)	0.399			
**HBsAg reduction from mid-pregnancy to delivery, log10 IU/mL**	1.456	(0.499,4.247)	0.491			
**ALT at baseline, U/L**	1.041	(0.970,1.118)	0.265			
**AST at baseline, U/L**	1.011	(0.943,1.084)	0.765			
**HBV DNA at baseline, log10 IU/mL**	0.604	(0.200,1.823)	0.371			
**HBsAg at baseline, log10 IU/mL**	0.043	(0.007,0.276)	0.001	0.051	(0.003,0.273)	0.010
**HBsAg at week12, log10 IU/mL**	0.188	(0.076,0.462)	<0.001	0.505	(0.090,1.811)	0.368
**HBsAg decline at week12, log10 IU/mL**	4.283	(1.511,12.141)	0.006	1.978	(0.552,11.143)	0.368
**ALT at week12 >2ULN**	1.482	(0.394,5.579)	0.560			
**HBsAg at week24, log10 IU/mL**	0.161	(0.063,0.413)	<0.001	0.214	(0.033,0.616)	0.022
**HBsAg decline at week24, log10 IU/mL**	4.202	(1.885,9.368)	<0.001	4.682	(1.624,30.198)	0.022
(B) Prediction model	AUC	Cut-off point	Sensitivity	Specificity	Youden index	P
**HBsAg at baseline, log10 IU/mL**	0.924	2.260	0.957	0.708	0.665	0.001
**HBsAg at week24, log10 IU/mL**	0.969	0.590	0.957	0.875	0.832	<0.001
**HBsAg decline at week24, log10 IU/mL**	0.863	1.080	0.875	0.870	0.745	<0.001

OR, odds ratio; CI, confidence interval; NAs, nucleos(t)ide analogs; AST, aspartate aminotransferase; ALT, alanine aminotransferase; HBV, hepatitis B virus; HBsAg, hepatitis B surface antigen; ULN, upper limit of normal; AUC, area under curve.

P<0.05 was considered statistically significant.

**Figure 3:**
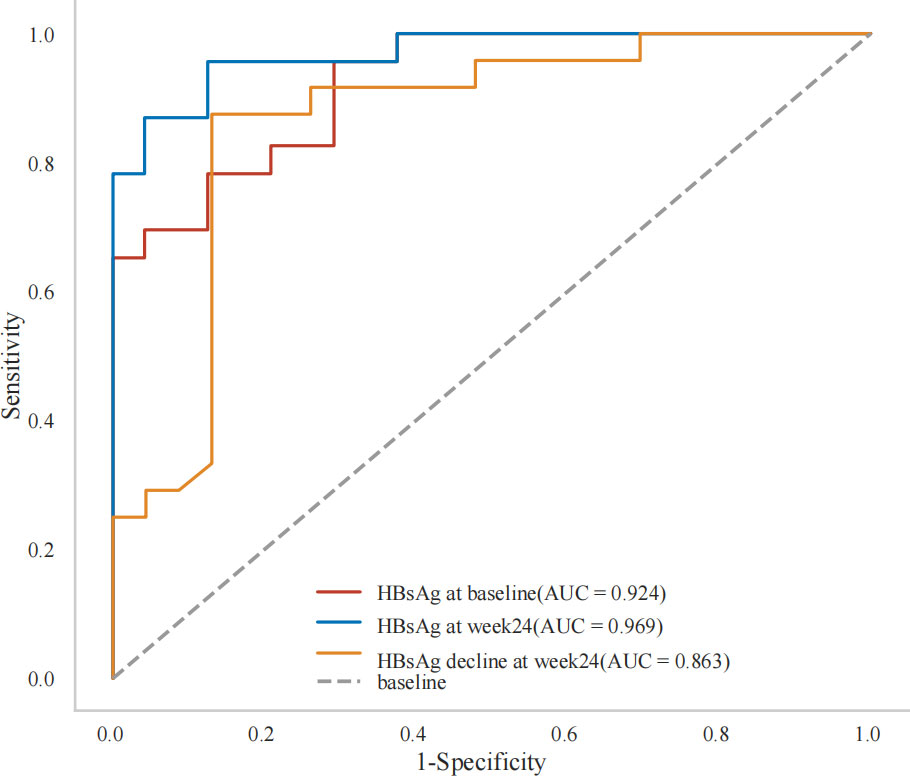
Prediction of HBsAg loss at week 48 in patients who received Peg-IFN treatment.

Furthermore ROC analysis also showed that baseline HBsAg<182 IU/mL,HBsAg at week24 < 4 IU/mL and HBsAg decline at week24>12IU/mL were all independent predictors of a functional cure (**Table 3B**, **Figure 3**).

### 3.4 Safety

No serious adverse events occurred among the patients in the Peg-IFN (+) group. The most common adverse events included fever (31/47, 65.96%), fatigue (21/47, 44.68%), alopecia (20/47, 42.55%), decreased appetite (40/47, 85.11%) and neutropenia (38/47, 80.85%) (**Table 4**). A total of 17 out of 47 (36.17%) patients treated with Peg-IFN alpha-2b had anemia, with hemoglobin levels were between 90-110 g/L. Among patients with thrombocytopenia, 5 out of 47 (10.64%) had platelet counts <75×10^9^ but >50×10^9^. The patients above were not given the specific treatment and relieved after the end of the Peg-IFN alpha-2b therapy. Among patients with neutropenia, 18 out of 47 (38.3%) had absolute neutrophil counts <1.0×10^9^/L, with 6 (12.77%) patients having counts <0.75×10^9^/L. The counts were improved to >0.75×10^9^ for all six patients after treatment with granulocyte stimulating factor (GSF).

**Table 4:** Adverse effect of Peg-IFN alpha-2b.

Type	N (%)	Treatment	Outcome
Body as whole			
Fever	31 (65.96)	Acetaminophen or no treatment	Relief/spontaneous remission
Headache	12 (25.53)	None	Self-relief
Fatigue	21 (44.68)	None	Self-relief
Myalgia	15 (31.91)	None	Self-relief
Dermatological			
Rash	1 (2.13)	None	Self-relief
Pruritus	2 (4.26)	None	Self-relief
Alopecia	20 (42.55)	None	Self-relief
Digestive tract			
Decreased appetite	40 (85.11)	None	Self-relief
Diarrhea	7 (14.89)	None	Self-relief
Nausea	3 (6.38)	None	Self-relief
Hematological			
Anemia	17 (36.17)	None	Relief after discontinuation of Peg-IFN alpha-2b therapy
Neutropenia	38 (80.85)	GSF or no treatment	Relief after GSF therapy or discontinuation of Peg-IFN alpha-2b therapy
Thrombocytopenia	7 (14.89)	None	Relief after discontinuation of Peg-IFN alpha-2b therapy
endocrine system			
Abnormal thyroid function	3 (6.38)	None	Relief after discontinuation of Peg-IFN alpha-2b therapy

Peg-IFN alpha-2b, pegylated interferon alpha-2b; GSF, granulocyte stimulating factor.

Among three patients with abnormal thyroid function, one patient (thyroid stimulating hormone[TSH] 0.21 uIU/mL) had already achieved HBsAg seroconversion before thyroid abnormalities were detected. Her abnormal thyroid function resolved after the Peg-IFN alpha-2b therapy stopped. Another patient (TSH 0.11 uIU/mL) was observed that the thyroid function normalize after 5 weeks following of suspending Peg-IFN therapy. The third patient had subclinical hypothyroidism (TSH 5.05 uIU/mL) and the thyroid function returned to normal after 6 weeks of interferon suspension, prompting us to continue the original Peg-IFN dose. None of the three had associated clinical symptoms.

## 4 Discussion

Peg-IFN alpha therapy is currently the main approach for achieving functional cure, but its effectiveness varies widely in different people. Postpartum mothers can undergo significant changes in the activity of immune cells and immune factors due to hormonal shifts and immune system reconstitution, potentially making this period a good opportunity to achieve functional HBV cures for mothers with chronic HBV infection and low HBV DNA levels and low HBsAg levels (Liu et al., 2017; Feng et al., 2023; Zhang et al., 2023). However, current guidelines (Chinese Society of Infectious Diseases, Chinese Medical Association; Chinese GRADE Center, 2019; Kumar et al., 2022) on whether and how to use antiviral therapy postpartum remain contentious. Most studies on the functional cure induced by interferon exclude this population due to the challenges of managing and following them up during this physiological state. Limited data have been reported on the functional cure of Peg-IFN alpha-2b treatment for postpartum HBeAg-negative women with chronic HBV infection. This study aimed to evaluate the effectiveness and safety of Peg-IFN alpha-2b in this group, providing data to support that postpartum women with HBV could be an advantageous population for a functional cure with Peg-IFN alpha.

A total of 51.06% and 40.43% of the patients in this study achieved HBsAg loss and seroconversion, respectively, at 48 weeks after treatment with Peg-IFN alpha-2b. Even after PSM matching, these rates remained significantly higher than in the group that did not receive Peg-IFN alpha-2b. The functional cure rate among the HBeAg-negative HBV mothers after Peg-IFN application was notably higher, surpassing the 10-40% cure rate seen in similar non-postpartum populations in other studies (Broquetas et al., 2020; Wu et al., 2020; Chu et al., 2022).

At present, the mechanism that induces postpartum HBsAg loss is not fully understood. Zhang et al. (2023) described the potential mechanisms that immune tolerance during the pregnancy could be broken due to the decrease of estrogen, progesterone and HCG after delivery. The immune system was transformed from Th2 and Treg dominant in pregnancy to Th1 and Th17 dominant after delivery. Natural killer (NK) cells, helper T (Th)cells, CD8+ T cells and multiple cytokines such as IFN-r, IL-2 and TNF-α could be involved (Joshi et al., 2017; Huang et al., 2021; Zhang et al., 2023). The change of immune system could last for at least 1 year after delivery (Samadi Kochaksaraei et al., 2020; Zhang et al., 2023). Studies (Huang et al., 2021; Song et al., 2022) also observed the CD4+ T cell and CD8+ T cell activation in patients with postpartum flares. There is an elevated frequency of CD8+ T cells expressing perforin and granzyme B. Among HBV-infected pregnant women, about 10-30% (Giles et al., 2015; Chang et al., 2018; Wang et al., 2022b; Zhang et al., 2023) of pregnant women have postpartum hepatitis, which may be related to reactivation in the immune system. Thus, the postpartum period might be an opportune time to achieve a functional cure for HBV.

Additionally, none of the 47 individuals included in the study experienced serious adverse events, demonstrating both the safety of Peg-IFN alpha-2b in maternity care and the potential effectiveness of treating HBeAg-negative HBV mothers in terms of achieving functional cure.

After further analysis of the factors influencing the maternal functional cure of HBV, we found a robust association between lower HBsAg levels at baseline and a rapid decline in HBsAg levels early in the treatment process with heightened rates of HBsAg loss at 48 weeks. Analogous observations have occurred in other non-postpartum cohorts (Ning et al., 2014; Hu et al., 2018; Chu et al., 2022).

We found that women with abnormal postnatal liver function, especially those with postnatal liver function >2ULN, which is postpartum flare, were more likely to achieve functional cure with Peg-IFN alpha-2b. Indeed, previous studies (Buster et al., 2009; Liaw et al., 2011) have found that patients with higher liver function at baseline have better outcomes with Peg-IFN, but it is important to note that the most HBV-infected mothers were close to or in immune tolerant carrier state or inactive HBV carrier state prior to pregnancy. These populations are characterized by consistently normal ALT or AST levels and infrequent pathological changes in liver tissue. They are in a relatively stable condition, with a low likelihood of liver function abnormalities (Chu and Liaw, 2007; Chang and Liaw, 2014). Morever, the timing of occurrence is uncertain as well. It is difficult to identify the onset of elevated liver function to start interferon therapy. The incidence of postpartum hepatic flare (Giles et al., 2015; Chang et al., 2018; Wang et al., 2022b; Zhang et al., 2023) is much higher than the incidence of hepatic abnormalities in the general population, with at least about 7.3% incidence of postpartum flare even in the HBeAg-negative mothers (Giles et al., 2015). Moreover, the time of occurrence of postpartum flare was more concentrated, mostly within 3 months postpartum (Wang et al., 2022b). This also means that we can identify those who can be clinically cured by interferon through regular monitoring of liver function in the postpartum population during the first 3 months after delivery.

Postpartum flare may represent an opportunity to achieve a functional cure.

However, this study had several limitations. Firstly, as this was a retrospective study, data were missing regarding pregnancy and the number of subjects included was relatively small. Recent studies (Chu et al., 2007; Chu et al., 2013; Tuna et al., 2024) have suggested a potential correlation between lipid metabolism and HBsAg seroclearance in HBV patients. However, the lack of data on changes in lipids and weight hindered our ability to delve deeper into this correlation. Secondly, no clear definition of postpartum in the literature, and after reviewing the literature (Liu et al., 2017; Feng et al., 2023; Zhang et al., 2023), we included women before 18 months postpartum in this study. In addition, we found that the relationship between the time of initiation of treatment in the postpartum period and the rate of HBsAg loss was not significant, which could be due to the limited sample size, this finding remains to be further confirmed by prospective large-data studies. Therefore, we suggest conducting a broader, prospective study involving multiple centers and a larger pool of patients, commencing from early pregnancy, to analyze various potential influences during pregnancy and postpartum.

## Data availability statement

The raw data supporting the conclusions of this article will be made available by the authors, without undue reservation.

## Ethics statement

The studies involving humans were approved by the Ethics Committee of the First Affiliated Hospital of Xi’an Jiaotong University. The studies were conducted in accordance with the local legislation and institutional requirements. The participants provided their written informed consent to participate in this study.

## Author contributions

WZ: Data curation, Formal analysis, Investigation, Writing – original draft, Writing – review & editing, Methodology, Software. LY: Data curation, Investigation, Writing – original draft. YZ: Data curation, Investigation, Writing – review & editing. LS: Project administration, Supervision, Writing – review & editing. YH: Project administration, Resources, Supervision, Writing – review & editing. TC: Conceptualization, Funding acquisition, Project administration, Resources, Supervision, Writing – review & editing, Writing – original draft. JZ: Conceptualization, Methodology, Supervision, Writing – review & editing, Writing – original draft.

## Glossary

**Table:**

HBeAg	hepatitis B e antigen
Peg-IFN alpha-2b	pegylated interferon alpha-2b
HBsAg	hepatitis B surface antigen
PSM	propensity score matching
HBV	hepatitis B virus
HCC	hepatocellular carcinoma
HBsAb	hepatitis B surface antibody
CHB	chronic hepatitis B
cccDNA	covalently closed circular DNA
MTCT	mother-to-child transmission
NAs	nucleos(t)ide analog
EMR	Electronic Medical Records
ULN	upper limit of normal
SDs	standard deviations
IQR	interquartile range
AST	aspartate aminotransferase
ALT	alanine aminotransferase
OR	odds ratio
CI	confidence interval
ASALD	American Association for the Study of Liver Diseases
ROC	receiver operating characteristic curve
AUC	area under curve
GSF	granulocyte stimulating factor
TSH	thyroid stimulating hormone
